# Simulation-based neonatal resuscitation training of medical students: is Peyton’s 4-stage approach more effective than traditional 2-stage technique?

**DOI:** 10.1186/2194-7791-1-S1-A4

**Published:** 2014-09-11

**Authors:** Erol Tutdibi, Ludwig Gortner, Thomas Volk, Erik Reus

**Affiliations:** Department of Pediatrics and Neonatology, Intensive Care Medicine and Pain Therapy, University of Saarland, 66421 Homburg/Saar, Germany; Department of Anaesthesiology, Intensive Care Medicine and Pain Therapy, University of Saarland, 66421 Homburg/Saar, Germany

Resuscitation of newborns is a critical procedure and requires refined skills. The contents of resuscitation guidelines and their implementation should be crucial in education of medical students. The traditional 2-stage (“see one, do one”) instruction is theory-loaded and does not provide sufficient hands-on training needed to give trainees the necessary practical experience or confidence in clinical skills. We aimed to evaluate whether simulation-based skill training by Peyton’s 4-step approach can improve the performance of medical students in neonatal resuscitation compared to traditional front-side 2-step teaching. In a prospective trial design, 49 fourth-year medical students were randomized in traditional and 4-stage groups. According to the “see one, do one” principle in the traditional group the teacher at first explained the ERC-algorithm and demonstrated the neonatal resuscitation procedure before the practitioners could train on manikins. In the “Peyton” group students were divided in small-groups with 4 trainees each and received 4-step approach training. Three days after the initial neonatal resuscitation training all students passed a standardized simulation-based scenario with newborn manikins to assess the effect of both teaching methods by an objective structured clinical examination (OSCE) and binary checklist. We modified the OSCE with a practical (pOSCE, maximal score 118) and a theoretical part (tOSCE, maximal score 74). Students in 4-stage group scored significantly higher than traditional trained participants: 113±5 vs. 68±23 (pOSCE) and 66±5 vs. 40±23 (tOSCE), all p<0.001. In conclusion, our study demonstrates that simulation-based training by Peyton’s 4-stage approach is effective to increase the performance of medical students in neonatal resuscitation.

